# Herpes Simplex Virus Esophagitis in an Immunocompetent Patient

**DOI:** 10.7759/cureus.44668

**Published:** 2023-09-04

**Authors:** Raj Shah, Stuti Patel, Richard Henriquez, Jignesh Parikh, Amar Mandalia

**Affiliations:** 1 Internal Medicine, University of Central Florida College of Medicine / Hospital Corporation of America Healthcare Graduate Medical Education, Orlando, USA; 2 Internal Medicine, Western Reserve Health Education, Trumbull Regional Medical Center, Warren, USA; 3 Pathology, Orlando Veterans Affairs Medical Center, Orlando, USA; 4 Gastroenterology, Orlando Veterans Affairs Medical Center, Orlando, USA

**Keywords:** rare, anabolic steroid, immunocompetent, esophagitis, herpes simplex virus

## Abstract

Esophagitis due to herpes simplex virus (HSV) infection is a rare entity in the immunocompetent population. It is usually seen in immunocompromised hosts, those with human immunodeficiency virus (HIV) infection, malignancies, and patients on immunosuppressive medications.

We present a case of a young immunocompetent man with anabolic steroid use who presented with esophagitis symptoms found to be from HSV infection. So far, the use of corticosteroids has been reported as a predisposing factor for HSV esophagitis in immunocompetent hosts in multiple case reports. However, our case suspects that transient immunosuppression with similar medication can cause HSV esophagitis in otherwise immunocompetent hosts.

## Introduction

Esophagitis from the herpes simplex virus (HSV) is well-known and a potential complication in immunosuppressed populations. However, only a few cases have been reported in otherwise healthy and immunocompetent patients [1-4]. When seen in immunocompetent patients, HSV esophagitis is typically reported in males in their 30s [5]. Due to transient immunosuppression, immunocompetent patients may be susceptible to conditions typically seen in immunosuppressed patients. While anabolic steroids have been linked to immunosuppression [6], their association with HSV esophagitis has not been established. Herein, we present an interesting and rare case of HSV esophagitis in an immunocompetent host who reported intermittently using non-prescription anabolic steroids.

## Case presentation

**Figure 1:**
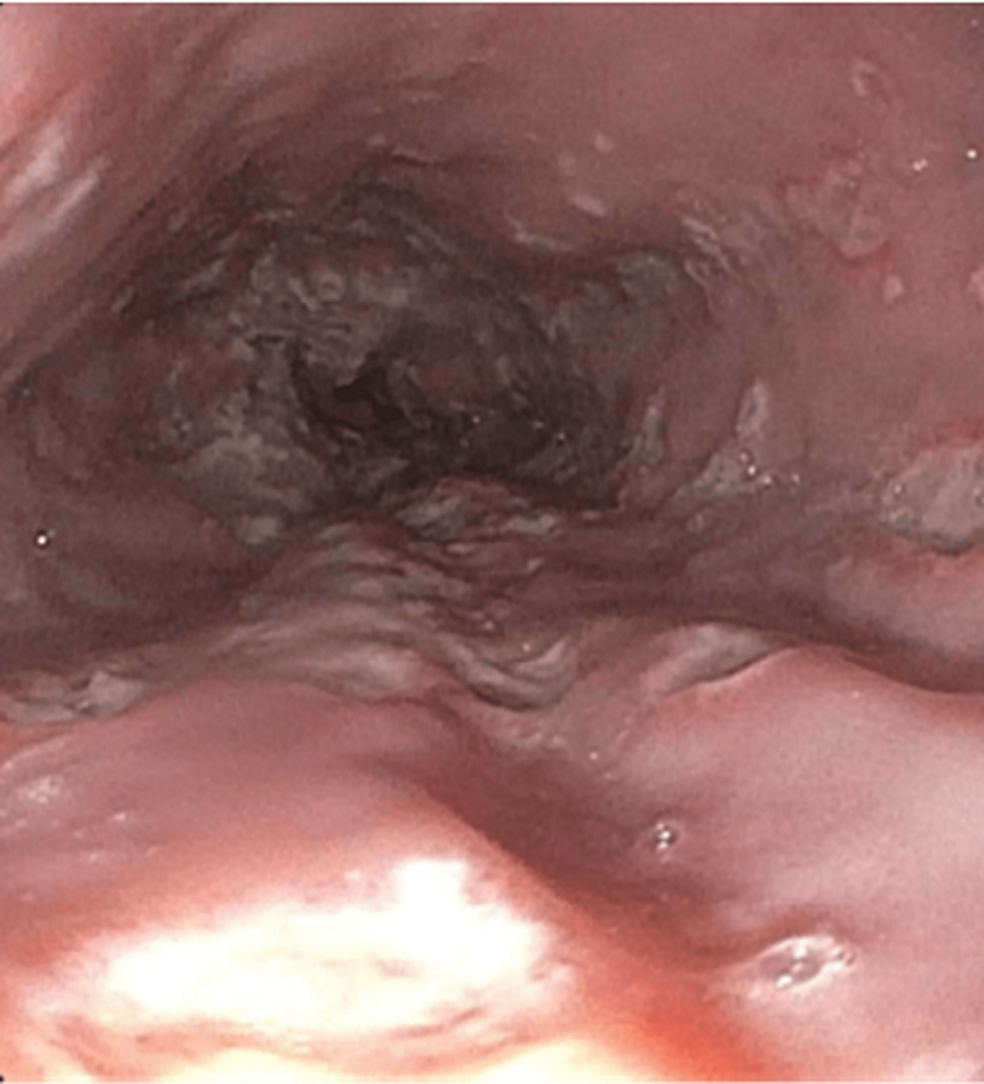
Esophagogastroduodenoscopy (EGD) shows ulcerative esophagitis in the lower third of the esophagus.

A 27-year-old male with no past medical history presented to the hospital complaining of burning pain extending from the throat to the epigastric region associated with nausea, bilious emesis with streaks of bright red blood, and odynophagia for five days. The patient mentioned experiencing generalized body aches but did not report any other symptoms, such as cough, diarrhea, constipation, chest pain, palpitation, back pain, headache, dizziness, or recent episodes of fever. He had been taking over-the-counter calcium carbonate regularly without significant relief. He did not have any previous esophagogastroduodenoscopy (EGD) or colonoscopy. He reported intermittent use of anabolic steroids (last use being two weeks before presentation) and occasionally smoking marijuana (last use being three weeks before presentation). He had been self-administering testosterone, which he procured from outside the country without a medical prescription. Based on his online search, he elected to inject himself with a dosage of up to 150 mg weekly. Computed tomography (CT) of the abdomen and pelvis with contrast was negative for any acute processes. The patient underwent EGD, which showed many cratered, punched-out esophageal ulcers with no active bleeding or stigmata of recent bleeding at 35-43 cm from the incisors (Figure 1).

**Figure 2:**
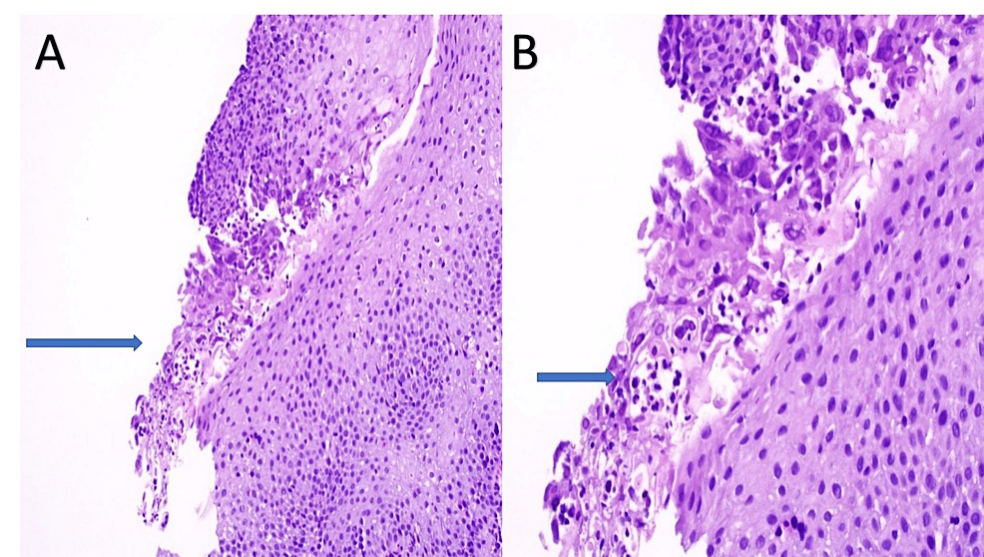
Squamous epithelium shows herpes simplex virus (HSV) infection consisting of multinucleation, nuclear molding, and margination (A and B).

**Figure 3:**
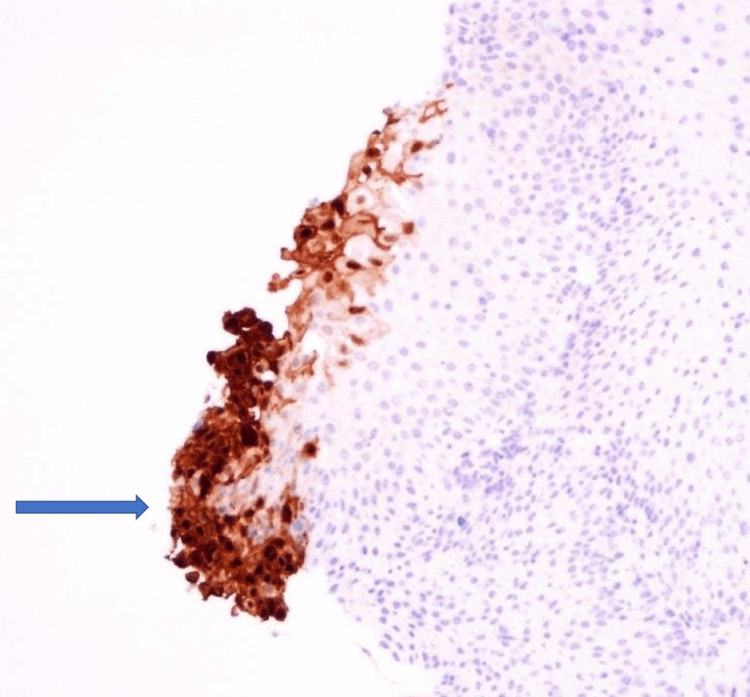
Immunostaining for the herpes simplex virus (HSV) highlights positive cells.

Biopsies were taken with cold forceps for histology and HSV and Epstein-Barr virus (EBV) staining. Herpetic esophagitis with ulcer was confirmed with immunostaining (Figures 2A, 2B, 3).

Typical cytomegalovirus (CMV) and periodic acid-Schiff (PAS) stains were negative. Serology testing was initially negative for HSV-1 immunoglobin M (IgM)/IgG, but two months later, IgG was positive. Hepatitis B, hepatitis C, chlamydia, gonorrhea, syphilis, HSV-2 IgM/IgG, and HIV were negative. Ceruloplasmin and alpha-1 antitrypsin (AAT) were within normal limits, and antinuclear antibodies (ANA) and anti-mitochondrial antibodies (AMA) were negative. The patient was treated with pantoprazole, sucralfate for eight weeks, and acyclovir for 10 days. At the three-month follow-up in the gastroenterology clinic, the patient reported a resolution of symptoms, including odynophagia.

## Discussion

Herpes simplex virus esophagitis is frequently described in immunocompromised hosts and rarely in immunocompetent patients. It can occur as a primary infection or reactivation and is characterized by acute-onset systemic manifestations and extensive erosive-ulcerative involvement of the mid-distal esophagus [1]. The mean age of incidence is 35 years (with male predominance) [1,5]. Most cases of HSV esophagitis are due to HSV-1, but a few case reports of HSV-2 esophagitis have been described [5,7]. Patients usually have a prodromal systemic manifestation of fever, nausea, or vomiting before manifesting esophageal symptoms. The most common symptoms are odynophagia, dysphagia, heartburn, epigastric pain, or chest pain. Concurrent oropharyngeal lesions are uncommon in immunocompetent hosts, but immunocompromised hosts usually have co-existent herpes labialis or oropharyngeal ulcers [1,8,9]. In our case, an immunocompetent male presented with generalized body aches, epigastric pain, nausea, vomiting, and odynophagia with no fever before or during hospitalization.

The diagnosis of HSV esophagitis is usually based on endoscopic findings confirmed by histopathological examination. Endoscopic findings include friable mucosa with numerous well-circumscribed ulcers smaller than 2 cm, most commonly in the distal esophagus [1]. The margin of an ulcer, where viral cytopathic activity is most likely to be noticed, should be the location of biopsies. Multinucleated giant cells with ground-glass nuclei and eosinophilic inclusions are examples of histologic findings. Additionally helpful may be an immunohistochemical analysis for HSV glycoproteins [1,8]. Serology usually shows an acute infection pattern (positive IgM antibody and negative IgG antibody) for HSV type 1 (more frequently) or HSV type 2 infection, with possible seroconversion up to three to four weeks later [1]. In our case, the patient underwent EGD, showing characteristic ulcers in the distal esophagus. Histopathology, as well as immunostaining, were diagnostic of HSV. The patient’s serology was initially negative for HSV-1 IgM and IgG, but he later seroconverted. Testing for sexually transmitted infections, including HIV, was negative.

In immunocompetent patients, spontaneous remission typically happens within one to two weeks. However, some patients may respond more quickly to a brief course of oral acyclovir 200 mg five times per day or 400 mg three times per day for seven to 10 days [10]. To date, no trials have been done for using acyclovir in HSV esophagitis in immunocompetent patients, but prompt symptom resolution has been reported [11]. Our patient was treated with acyclovir along with proton pump inhibitors and sucralfate.

Multiple case reports have been published linking the use of corticosteroids with the development of HSV esophagitis, even in immunocompetent patients [12-14]. This may indicate that even in patients with intact immunity, transient immunosuppression can predispose them to HSV infection of the esophagus. However, no reports have been published about HSV esophagitis in those using anabolic steroids [6].

## Conclusions

We present a case of a young male with anabolic steroid use who complained of odynophagia. Upon conducting an endoscopy, we discovered ulcers in the lower esophagus caused by the herpes simplex virus. Given that herpes simplex virus esophagitis is usually seen in immunocompromised patients, common causes of immunosuppression, including HIV, were ruled out. Although there are no prior reports of this condition being linked to anabolic steroid use, we suspect that the patient's use of these steroids may have made him more vulnerable to the virus. While the causation cannot be established, it would be prudent for physicians to be mindful of anabolic steroid use as a potential cause of pathologies typically seen in immunocompromised patients. Additionally, it emphasizes the importance of reporting future cases of patients who use anabolic steroids and exhibit conditions typically found in immunosuppressed patients.
